# Preventing sensationalistic science and fake news about substance use

**DOI:** 10.1186/s13011-018-0148-3

**Published:** 2018-03-12

**Authors:** Stephan Arndt, De Shauna Jones

**Affiliations:** 1 Iowa Consortium for Substance Abuse Research and Evaluation, University of Iowa, 100 MTP4, Iowa City, IA 52245-5000 USA; 2 Department of Psychiatry, Carver College of Medicine, University of Iowa, 200 Hawkins Drive, Iowa City, IA 52242 USA; 3 Department of Biostatistics, College of Public Health, University of Iowa, 145 N. Riverside Drive, Iowa City, IA 52245-5000 USA

An accurately informed public is vital to creating rational substance use, prevention, and treatment policies. Unfortunately, the general public neither has the training nor the time to conduct intensive investigations. Most people get their information via the news media, newsletters, and as sound bites. People often accept what they read as true, especially if it comes from a reasonably reputable source and do not question the information however astounding or alarming. It is easy to forget the adage, “Extraordinary claims require extraordinary evidence.” To make matters worse, people may tend to repeat the more remarkable information, regardless of how accurate it is.

I recently investigated two remarkable pieces of information regarding substance use issues. These both pertained to opioids, but might be equally enlightening no matter what substance was involved.

## Case 1

I recently read that 14–22% of all pregnancies in the United States were complicated by use of prescription opioid medications. This was in a newsletter [1] referencing a paper in the BMJ [2]. The newsletter description goes on to say that an infant showing signs of opioid withdrawal is born every 25 min. Despite their proximity, the two statistics represent different things.

A reader might easily take this statement to suggest that about one in five births are complicated by opioid use. We were reasonably surprised by that and so read the original article. Interestingly, results of the referenced paper by Huybrechts et al. [2] indicated no such thing. Instead, the statement in the newsletter referencing 14 to 22% was in Huybrechts et al’s [2] introduction and was attributed to two previous papers (reference numbers 3 and 4). One of the first source papers suggested that 14.4% of women were prescribed opioid medication at some point in their pregnancy [3] while the other reference found that 22% of Medicaid women were prescribed opioids at some point in their pregnancy [4]. Note that these first source references suggest the 9-month prevalence of receiving an opioid medication during pregnancy and do not propose that the resulting birth suffered opioid withdrawal or that there were any complications.

The article, referenced by the newsletter [2], found that only 2.3% of births from women receiving opioid medication during their last trimester suffered discernable withdrawal. Therefore, the actual percentage of births suffering from withdrawal would be 0.023 times either 0.14 or 0.22 depending on whether we used the first sources’ estimates of 14.4% or 22%. Either of these calculations result in an estimate of less than 1% (0.3312% or 0.506%) and not 14 to 22%. Given that the periods of prescription were different for the source papers (9 months) and the article reference by the newsletter (3 months), the prevalence of births complicated by opioids is likely lower.

The statement that the one infant is born every 25 min came from another study [5] suggesting that neonatal abstinence syndrome rose from 0.34% to 0.58% between 2009 to 2012. This is similar to the estimate in the previous paragraph, 0.3312% or 0.506%, but a far cry from the estimates in the newsletter [1] and its referenced paper’s introduction [2], "14-22% of pregnancies are complicated due to the use of prescription opioid medications". The only way this can be interpreted as a correct statement is to define any opioid medication taken at any time during pregnancy as a complication. If the initially referenced authors and newsletter were equating *any* opioid prescription as a complication, then the equivalence was not transparent.

## Case 2

This is more of a generic situation rather than a specific instance. While speaking at a local school about opioid issues in Iowa, a teacher asked me "How many pain pills did it take to cause irreparable brain damage? Is it one, two, four?".

While it is certainly a possibility that an opioid overdose might cause oxygen deprivation, which in turn could cause brain damage, I do not think that was what the teacher was asking. He was asking about the direct damage caused by an opioid. Clearly, people take opioids for pain management from injury and surgery or cancer pain. Sometimes, the course of opioid treatment is days, even weeks. There is no evidence for brain damage in these situations. I did find some threads in discussion boards that claimed that certain spices (e.g., turmeric) could reverse the brain damage from opioid use, but these were unsupported “junk science” comments.

The magazine, Scientific American, had an online article "Can HGH Reverse Brain Damage in Drug Addicts?" [6] This article starts out in the first paragraph with the statement that opiates destroy brain cells, with no supporting peer reviewed references. The article goes on to describe a study in the Proceedings of the National Academy of Sciences, which shows that mouse neuronal cells in a petri dish bathed in morphine tend to die off. However, when also exposed to synthetic growth hormone, the die off was attenuated [7]. Several studies suggest little or no effects of prescribed opioid use and cognition [8–10]. While there may be some evidence that opioids cause apoptosis in neonates [11] and that extensive use of illicit opioids are associated with cognitive issues, there is scant evidence that they cause cell death or “brain damage” in adults. These references do not address whether or not opioids cause anatomical brain damage. The long term and widespread use of opioids for surgeries might suggest otherwise. Thus, the wording in the title, although catchy, may be misleading.

There are several things authors, reviewers, and editors can do to prevent misunderstandings and fake news. The simplest is to watch for odd and surprising statistics presented anywhere in an article. Remarkable comments need scrutiny. Papers need to highlight the importance of their work, but they also need to remain trustworthy and should not exaggerate. Hidden presumptions should not be a part of scientific literature since scientific communication demands transparent and replicable evidence.

Manuscripts require precise and accurate language devoid of hidden agendas. Words such as “cognitive impairment”, “deficiency”, and “deficits” appear often in scientific literature with regard to opiates and medication assisted treatment (MAT) effects. Of course, these papers are seldom experimental designs that can assess causal effects. Furthermore, these terms sound far more “ominous” than noting that the people in the opioid use group or MAT group had lower performance than the comparison group. For example, the differences between groups on some measure of cognitive functioning should be called a “difference”. Consider two groups where Group A has lower scores than Group B. If Group A were opioid users in treatment and Group B were a community sample who were age and sex matched, an author might suggest that Group A had cognitive “deficits”. However, hidden assumptions suddenly show up if instead of selecting Group A based on their opioid use we selected these people based their skin color or ethnic background. The appropriate neutral word would be “difference”.

Stigma, fear, and exaggeration are poor motivators to improve science or policy relating to substance use issues. In fact, we as scientists and educators should be represent reasoned rationality rather than sensationalism. Exaggeration, hidden presumptions that misinform, and unstated agenda are the antithesis of scientific literature and represents “fake news” and “junk science”. Authors need to present facts clearly and transparently. Reviewers and editors, as the guardians of rational and transparent information, need to question authors’ about wording issues, presumptions, and remarkable statements.
